# A new airtight sampling method for sulfur hexafluoride (SF_6_) in groundwater

**DOI:** 10.1016/j.mex.2024.103120

**Published:** 2024-12-18

**Authors:** Raka Sunderland, Dan Larsen, Scott Schoefernacker, Deborah Leslie

**Affiliations:** University of Memphis Department of Earth Sciences 109 Johnson Hall Memphis, TN 38152, USA

**Keywords:** Groundwater tracers, Sampling, Sulfur hexafluoride, Urban areas, Electrical transformers, Airtight Sampling Method for SF_6_ in Groundwater

## Abstract

In the past 20 years, sulfur hexafluoride (SF_6_) has been considered a highly reliable tracer for assessing modern water (< 65 yrs old) in groundwater. However, modern-air contamination may introduce complications in interpreting data obtained using current sampling methods. A new airtight methodology isolates the sample from modern ambient air; thus, returning more reproducible and reliable results when compared to two traditional (air-sensitive and non-airtight) methods. The new airtight method returned results within 0.03–0.05 fmol/L SF_6_ of expected SF_6_ concentration for pre-modern waters (0.02–0.09±0.01 fmol/L). In contrast, the air-sensitive and non-airtight traditional methods returned results within 0.00–0.90 fmol/L and 0.02–1.21 fmol/L of the expected value, respectively. It is suspected that transformers in proximity to wells leak SF_6_ which subsequently partitions into the samples obtained using traditional air-sensitive and non-airtight methods, thus creating erratic SF_6_ results.

• Comparative analyses of a new airtight method with traditional methods were performed and returned lower SF_6_ concentrations with the new 2022 airtight method.
• The new airtight method shows reproducible low-level detection of SF_6_ concentrations and may reduce sampling error in production wells in urban areas that are co-located with transformers which are SF_6_-emitting sources.

## Graphical abstract

**Figure:**
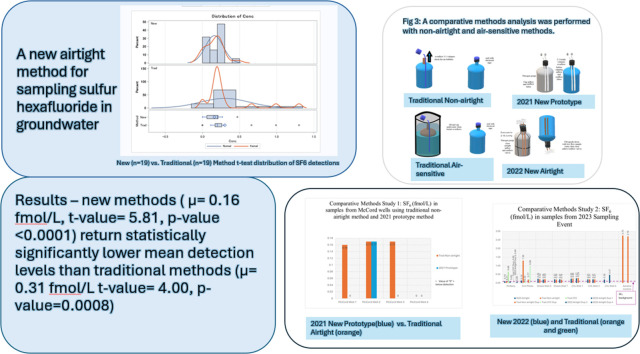

Specifications table

**Table:** 

Subject area:	Earth and Planetary Sciences
More specific subject area:	Environmental Sciences
Name of your method:	Airtight Sampling Method for SF_6_ in Groundwater
Name and reference of original method:	Traditional Non-airtight [1] Busenberg, E. and Plummer N. Dating young groundwater with sulfur hexafluoride: Natural and anthropogenic sources of sulfur hexafluoride. Water Resources Research 36(10) (2000) 3011–3030. Traditional Air-Sensitive [2] Plummer, N.L., Busenberg, E., and Cook, P. Use of Chlorofluorocarbons in Hydrology. (2006) International Atomic Energy Agency, Vienna Austria.
Resource availability:	R. Sunderland, Sulfur hexafluoride accumulation within groundwaters in Shelby County, Tennessee. M.S. Thesis, (2024) Department of Earth Sciences, University of Memphis, Memphis, TN.

## Background

The traditional method for sampling SF_6_ in groundwater, which is used to detect interaction with water <65 years old (modern water), uses an open-air, non-airtight bottle under the assumption that ambient air does not introduce significant amounts of SF_6_ to samples [1]. This method was used from 2009 onwards to sample wells in Shelby County, Tennessee, USA [[3], [4], [5], [6], [7], [8]]. Review of historical SF_6_ data from production and monitoring wells, sampled between 2009 and 2023 in Shelby County, Tennessee, shows samples have higher than the background SF_6_ concentration range (0.02–0.09±0.01 fmol/L) [1] for groundwater not affected by modern water leakage from shallow aquifer and/or surface water sources [7,9].

This research proposes that modern air can be introduced by sampling if using a non-airtight or air-sensitive method that allows ambient air into the sample bottle. This issue may be a larger problem in more recently sampled groundwater because SF_6_ levels are continuously rising in the Northern Hemisphere [10]. Sulfur hexafluoride concentrations above equilibrium North Hemisphere atmospheric concentrations have been found around landfills, Mg and Al smelting, and electrical utilities [11], which may contribute to higher concentrations in groundwater samples from wells co-located with such sources. This is particularly relevant to production wells in Shelby County, Tennessee, USA, where many wells are co-located (within the same wellfield) with utility pumping stations containing SF_6_ as an insulating gas in switchgear, circuit-breakers, and high-capacity electrical transformers. “High-capacity” is the term used in this study to refer to multi-transformer systems adjacent to large pumping stations that are used to supply lower voltages to densely populated areas.

Sulfur hexafluoride data analysis using the Closed Equilibrium (CE) Model [12] does not account for introduction of ambient air into the sample bottle when sampling, but only excess air entrained during aquifer recharge at the water table. Ambient air near wells co-located with electrical transformers is likely to have higher-than-expected concentrations of SF_6_ [3]. Partitioning of SF_6_ into groundwater during sampling and storage with non-airtight or air-sensitive methods may increase the dissolution of SF_6_, thereby causing modern-air contamination of samples. Although SF_6_ has a very low Henry's Solubility gas constant [13], even small additions can affect results from wells in Shelby County where expected concentration range is 0.02–1.29 fmol/L. Moreover, the low-level modern-air contamination may mask evidence of modern and pre-modern groundwater interaction, due to slow recharge times [14,15] and the depth of screened intervals (about 60–790 m) in the aquifers [16]. This study proposes an airtight method that aims to eliminate modern-air contamination during sampling and increase the precision and reliability of SF_6_ analysis in groundwater samples.

The historical sample dataset from 2009 to 2023 has concentrations that are frequently above 1.00 fmol/L, despite residence times for the Memphis aquifer in Shelby County based on ^14^C data of >1000–7000 years [14,15] and >10,000 years for Fort Pillow and McNairy aquifers [14,15], which should have non-detectable SF_6_. Tritium and SF_6_ ages contradict each other and, therefore, make interpretation of SF_6_ results more difficult, with SF_6_ indicating groundwater ≤40 years old in samples with ^3^H contents of 0.5 TU or near detection limits [17] indicating premodern water or water ≥65 years old [5].

**Fig. 1:**
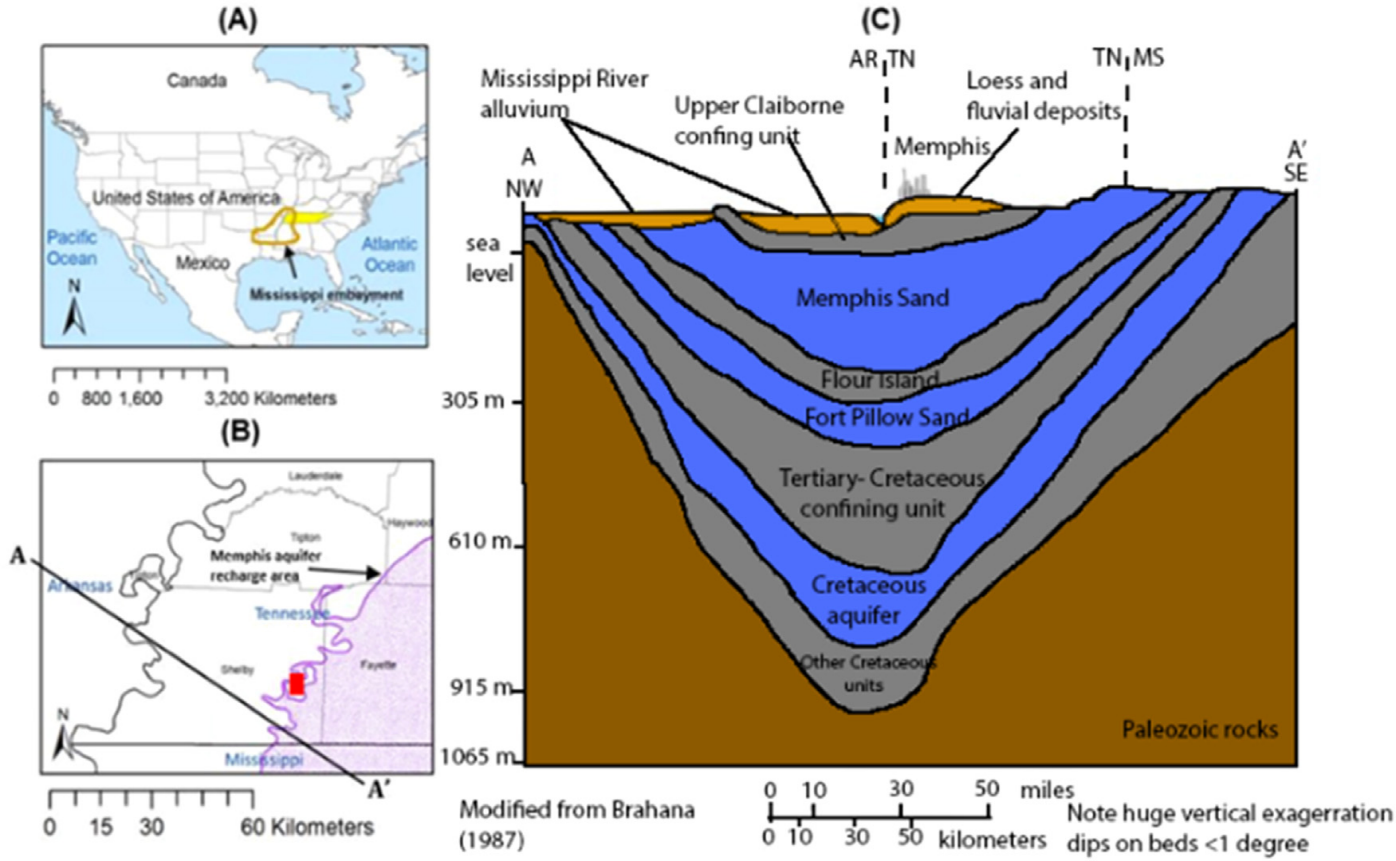
(A) Map showing study area in north Mississippi embayment; (B) The study area includes wellfields in Shelby County, Tennessee, and shows the unconfined area of the Memphis aquifer (recharge zone) in purple; (C) Cross section A-A’ showing the hydrostratigraphy beneath the study area including the shallow aquifer containing fluvial and alluvial deposits, Memphis aquifer, Fort Pillow aquifer, and McNairy (Cretaceous) aquifer [16].

The sampled wells in Shelby County draw from unconsolidated sedimentary aquifers (the Memphis, McNairy, and Fort Pillow aquifers (Fig. 1). Igneous and karst strata have been shown to naturally have higher terrigenic SF_6_ [9], but sedimentary aquifers have not shown this in the literature. Therefore, terrigenic formation of SF_6_ is not considered a major issue in this aquifer system.

**Fig. 2:**
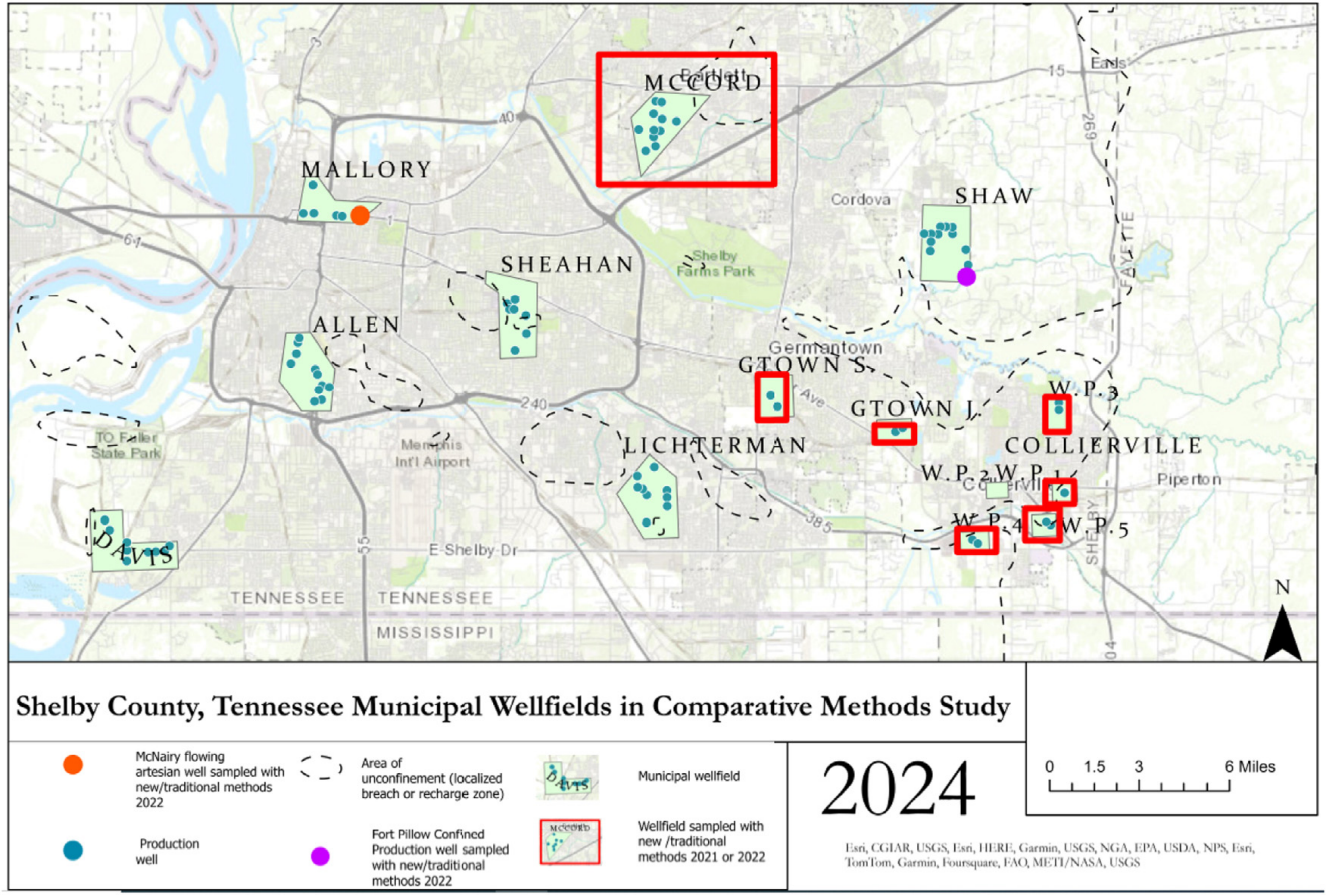
Comparative methods studies were performed on samples from a deep flowing artesian well (McNairy) in 2023, a deep confined production well (Fort Pillow) in 2023, and several wells located near breaches (McCord in 2022, Germantown in 2023) or recharge zones (Collierville in 2023).

Excess air is an issue for environmental tracers with low solubility like SF_6_ and ^3^H. Excess air can be introduced when well conditions are partially confined/unconfined and bubbles containing modern air in the water table or shallow aquifer dissolve due to increased hydrostatic pressure in the subsurface, causing artificially high concentrations of SF_6_ and ^3^H in the deeper, partially confined aquifer [12]. This can be an issue for Memphis aquifer groundwater samples since many samples come from wells locations in regions where there are breaches (partially missing or entirely absent confining layer) (Fig. 2). Since ^3^H sampling and analysis methods are equally prone to excess air issues [[4], [11], [12], [13]] but do not show higher-than-expected concentrations in the historical Shelby County dataset, it is proposed in this research that SF_6_ data from 2009 to 2023 has a component of modern-air contamination introduced during sampling, specifically in samples from wells near electrical transformers that have arc-quenching SF_6_ as an insulating gas. This type of contamination may have increased SF_6_ concentrations in samples while simultaneously not affecting ^3^H concentrations.

## Method details

This study tests if modern-air contamination during sampling using traditional air-sensitive [1] and non-airtight [2] methods can be reduced or eliminated by using newly developed 2021 prototype and improved 2022 airtight sampling methods by performing comparative studies. The sampled wells draw water from aquifers with long residence times based on ^14^C dating. The Memphis, Fort Pillow, and McNairy aquifers have residence times ranging from 1000 to >10,000 years [14,15], with the Fort Pillow and McNairy Aquifers being fully confined. Modern water mixing is not suspected from Fort Pillow and McNairy wells unless introduced during sampling or by leaky well construction (Fig. 1). The Memphis aquifer is confined in most locations with distributed semi-confined zones (breaches) in Shelby County and unconfined conditions in the eastern portion of Shelby County (Fig. 1, Fig. 2). The breaches and unconfined areas present water quality concerns in the urban settings, so accurate determination of groundwater age via SF_6_ is crucial to groundwater quality and vulnerability studies in Shelby County [[7], [8], [9], [15], [18]].

Samples were obtained in 2021 using the traditional non-airtight and 2021 prototype airtight sampling methods from production wells screened in the Memphis aquifer at the McCord wellfield (Fig. 2). A significant confining unit breach exists near the wellfield [18] that allows modern water to enter the Memphis aquifer and presents the potential for water quality issues [19]. Sulfur hexafluoride data may be useful for identifying wells most affected by modern water leakage. A pumping station with high-capacity electrical transformers is co-located within the McCord wellfield <1 km from MLGW Wells 1, 2, and 3 and <2 km from Well 4. All McCord wells sampled are screened 110–160 m below the land surface.

A test of robustness of the airtight procedure was completed in 2023. Samples were obtained using traditional non-airtight, traditional air-sensitive, and newly developed 2022 airtight procedures from two wells: 1) a monitoring well screened greater than 790 m deep in a flowing artesian aquifer (McNairy) that is sealed to the surface and was allowed to purge for seven days prior to sampling and 2) a production well screened at greater than 300 m in a fully confined aquifer (Fort Pillow) and allowed to purge for 24 h prior to sampling (Fig. 2). Both wells are <0.5 km from electrical transformers. Samples were also obtained in 2023 from two suburban wellfields (Collierville and Germantown) that are 0.1–4 km from pumping stations with high-capacity electrical transformers or pole-mounted distribution transformers (Fig. 2). These production wells are screened from 60 to 95 m in the Memphis aquifer, which is unconfined beneath parts of Collierville and fully confined beneath Germantown well locations.

It is expected that the Fort Pillow production well and McNairy well will have concentrations that reflect very old water sources (approximately 10,000 years old), that is, within ±0.01 of 0.02–0.09 fmol/L SF_6_ or background SF_6_ levels [1]. It is only expected that concentrations of SF_6_ will be higher if modern air-contamination affects sampling in these wells.

The McCord, Germantown and Collierville wells draw from the Memphis aquifer within or near the recharge zone (Germantown and Collierville) or locations near breaches (McCord) in the confining layer [[8], [9], [15], [18]]. This may cause SF_6_ concentrations to be significantly higher than background levels in these wellfields due to mixing of modern water with pre-modern aquifer water.

## Comparative methods analysis

### Traditional non-airtight method

**Fig. 3:**
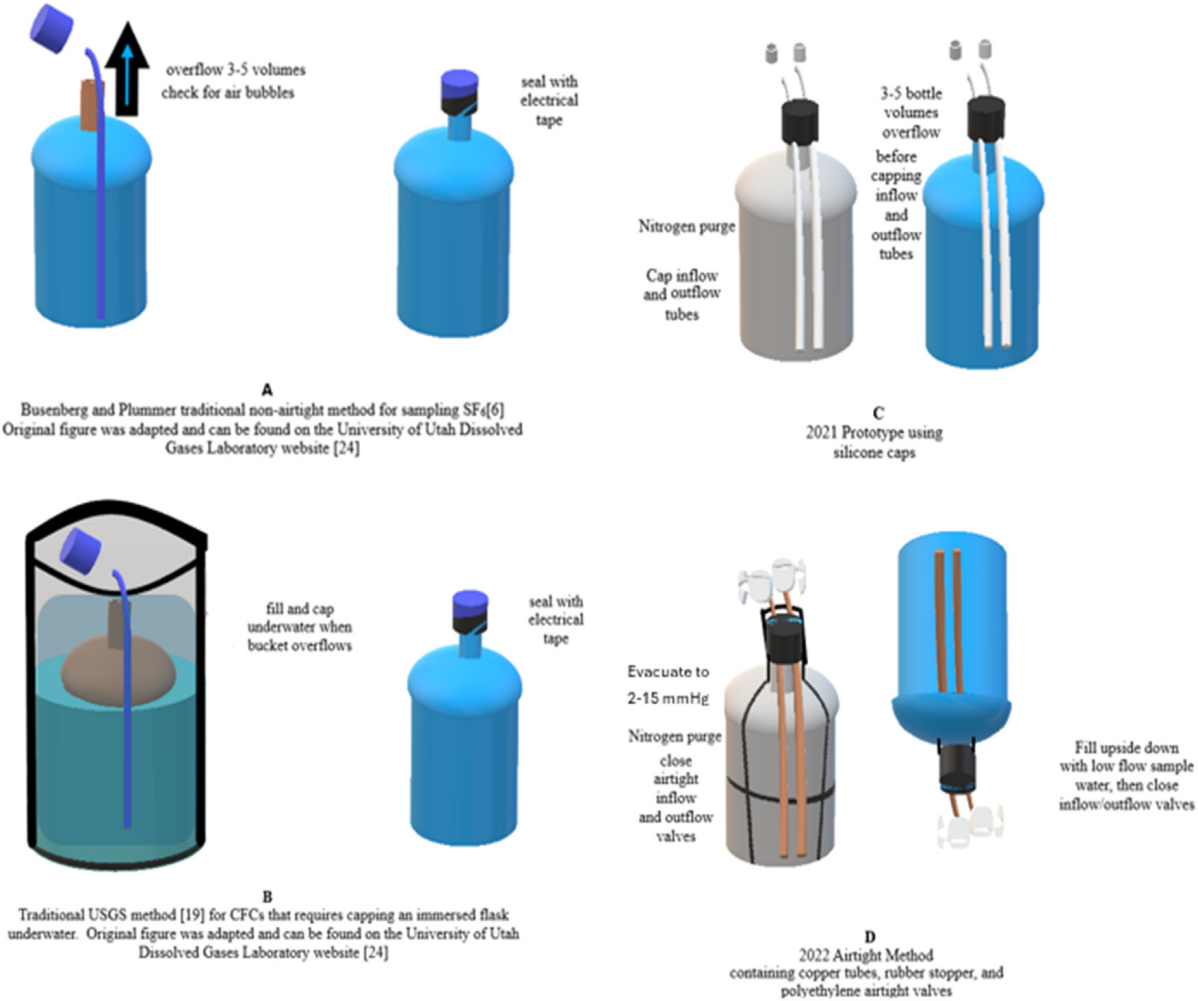
All methods used in comparative study including the traditional USGS non-airtight method (A), the traditional USGS method used to sample CFCs (B), the newly developed 2021 prototype method containing silicone in its assembly (C) and the newly developed 2022 airtight method containing no silicone in its assembly (D).

Busenberg and Plummer created the traditional non-airtight methodology used to sample and analyze SF_6_ from groundwater sampled from wells and Plummer et al. (2006) adapted it to create a more air-sensitive method for CFCs [[1], [2], [20]] (Fig. 3B). The original procedure (Fig. 3A) [1] requires taking one liter of groundwater directly from production and monitoring wells after three to five bottle volumes overflowed from a Boston round bottle. To minimize modern air contamination, a sampling tube is inserted into the bottom of the 1 L bottle during sampling. Air is then removed from the bottle by adding water to the conical seal-screw cap before capping an overflowing bottle. Finally, the bottle is checked for air bubbles before storing samples at a temperature of ∼ 4 °C to reduce increases in water volume. Sample bottle caps are then bound with electrical tape and shipped for chemical analysis via gas chromatography-electron capture detection (GC-ECD) to an equipped laboratory. Samples were analyzed at the University of Utah Dissolved Gas Laboratory and the U.S. Geological Survey (USGS) Reston Groundwater Dating Laboratory. The data were analyzed using Lumped Parameter modeling (LPM) after correcting SF_6_ concentrations for excess air, recharge temperature, and salinity using the Closed Equilibrium (CE) Model to obtain a groundwater age [[12], [17], [21], [22]].

### Traditional air-sensitive method

The traditional air-sensitive CFC method uses the same Boston round bottle as the traditional non-airtight SF_6_ sampling method but requires immersion of the sampling bottle in a bucket that is overflowing with groundwater and then capping of the bottle underwater [2] (Fig. 3B). This method is considered air-sensitive enough for CFCs, which are highly prone to modern air contamination [[11], [23]], and therefore is a suitable method to compare to the newly developed airtight method proposed in this study.

### 2021 Airtight prototype method

**Fig. 4:**
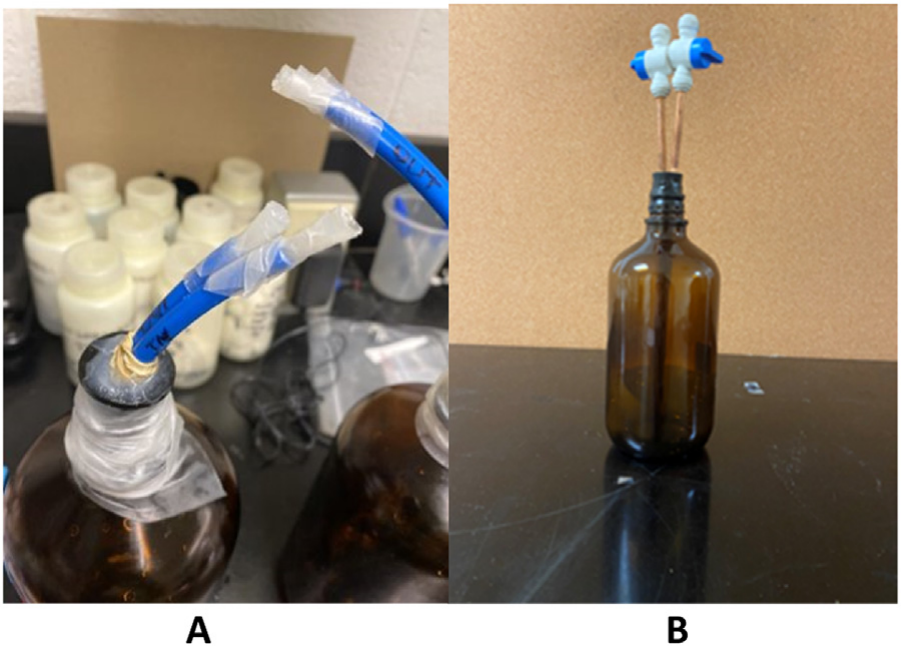
Reference pictures of (4A) 2021 Prototype Bottle Design and (4B) 2022 Airtight Bottle Design for SF_6_.

The airtight prototype procedure developed in 2021 requires one to purge the bottles with nitrogen about one hour before sampling. A modified lid keeps the nitrogen from diffusing. The lid is sealed by a silicone grommet affixed to two sampling tubes, an inflow and outflow tube. These tubes are sealed by silicone gel to the grommet and held onto the 1 L bottle with tightly bound parafilm (Figs. 3C, 4A).

**Fig. 5:**
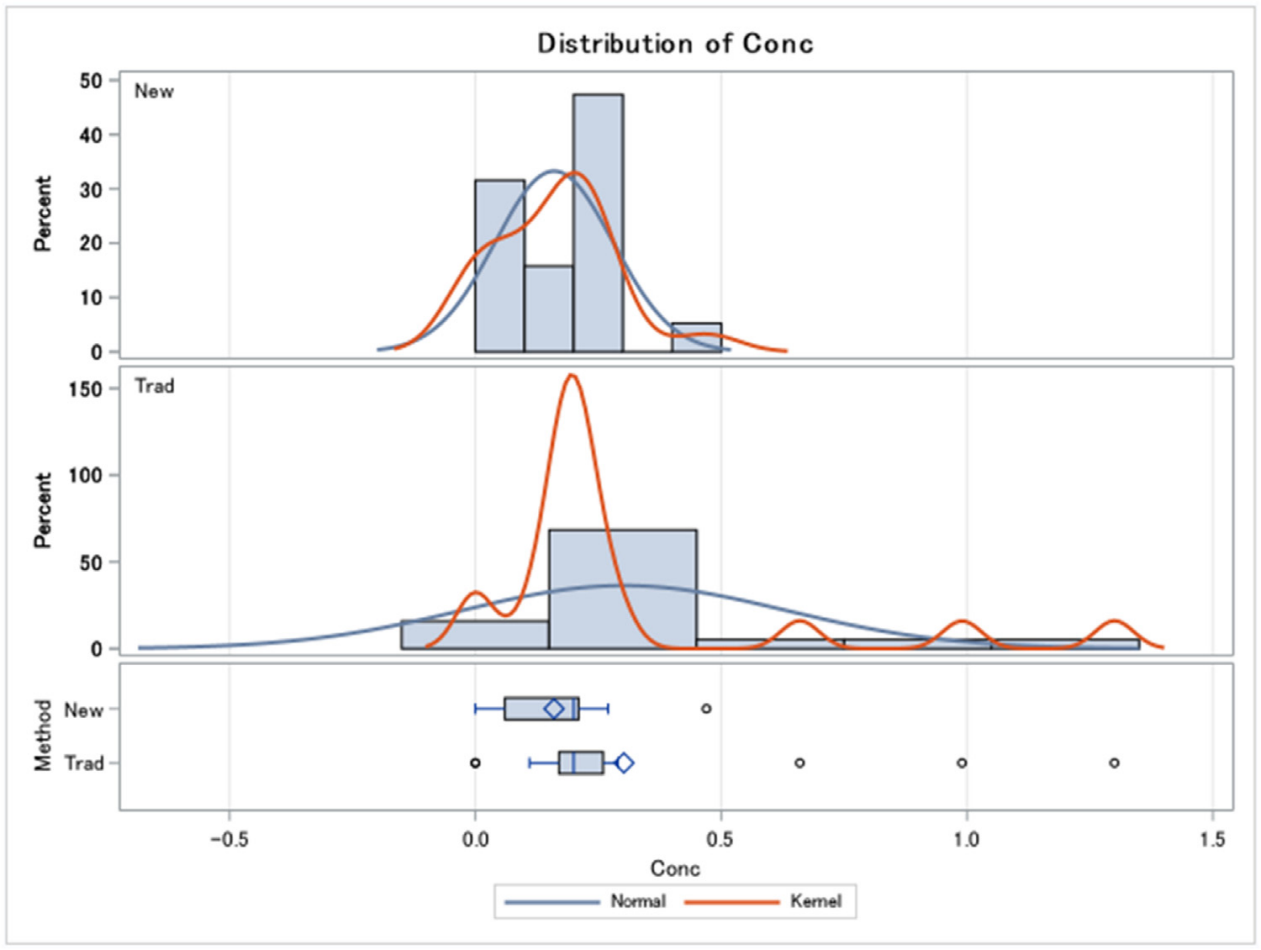
Comparison of spread of concentrations using New Methods (2021 and 2022) and Traditional Methods (Air-sensitive and Non-airtight).

The 2021 procedure requires each bottle to be purged for at least five minutes with nitrogen at low pressure, then the inflow and outflow tubes are sealed with silicone end caps before use in the field. Purging with nitrogen removes excess SF_6_ and oxygen in the bottle. The inflow tube connects to the well sampling tube in the field and three to five bottle volumes of water exit via the outflow tube before both the inflow and outflow tubes are capped. After removal of the modified lid, the bottle is sealed with a conical seal-screw cap filled with sampling water. The bottle is examined for air bubbles, stored in refrigeration, and then wrapped in electrical tape and shipped for chemical analysis to the University of Utah Noble Gases Lab. Tritium, a modern-water indicator in groundwater, was also sampled according to standard procedures and analyzed using the He-in growth technique [[13], [24], [25]]. The samples were analyzed at the University of Utah Dissolved Gases Lab in March 2022 and are shown in Table 1 and Fig. 5.

**Table 1:** Dataset includes Trad= traditional methods – includes Air-sensitive and Non-airtight methods and New = new methods – includes 2021 prototype and 2022 new airtight methods and sulfur hexafluoride concentrations in fmol/L.

	N	Mean (fmol/L)	Median (fmol/L)	Std. Dev. (fmol/L)	Var. (fmol/L)	Range (fmol/L)	t-stat	p-value
New Methods (2021 and 2022)	19	0.16	0.20	0.12	0.01	0.00–0.47	5.81	<0.0001
Traditional Methods (Air-sensitive and Non-airtight)	19	0.30	0.20	0.33	0.11	0.00–1.31	4.00	0.0008

### 2022 Airtight method

The new airtight procedure contains no silicone and when tested for airtightness, it maintains a pressure of 2–15 mmHg over 24 h. The construction procedure for the airtight sampling bottle is described in [8] (patent pending). Evacuate constructed bottle by opening inlet valve (either one) while keeping other valve closed. A five-minute evacuation using a standard vacuum pump assembly is recommended. Evacuation to near 0 mmHg can be tested by using a manometer attached to the vacuum assembly. A range of 2–15 mmHg pressures were read and maintained over one day in evacuated bottles. Although bottles are nearly evacuated, the risk of shattering remains. This risk is minimized by the reinforced design of the Boston round bottle and not allowing pressures to drop below 2 mmHg However, safety precautions should be used as with any low-pressure apparatus. It is recommended that all evacuation be done in a hood with a shatter shield in place.

Nitrogen-purge the bottle by connecting it to the inlet and opening the outlet valve for five minutes. It is important to use low pressure 99.99 % SF_6_-free nitrogen for purging. We recommend that the outlet valve is closed first, then the inlet valve, followed by shutting the low-pressure nitrogen source off to maintain a steady flow of nitrogen through the purge. This step is essential for creating an airtight, ambient air-free environment in the flask.

### Procedure for airtight flask use in the field

Setup ¼-inch diameter polyethylene tubing from well spigot to inlet valve before opening valve. Follow all procedures for USGS traditional non-airtight well sampling including purging well until geochemical data are stable [17]. The bottle should be turned upside down and water pressure should be low before opening the inlet valve to prevent bubbling and stripping of water samples. Open the inlet valve and keep the outlet valve closed until the bottle is at least half full. This forces all air to the bottom of the bottle where the copper wire can effectively remove any air bubbles when the bottle is nearly filled. Open the outlet valve when the bottle is at least ½ full and fill until no air bubbles are visible. Any air bubbles can be removed by gently rotating the bottle while it is upside-down to nudge bubbles into the copper wire where they can effectively be removed via the outlet valve. Close the outlet once all air bubbles have been effectively removed. Close inlet valve. Then remove ¼-inch diameter polyethylene tubing from inlet and outlet valves and place airtight sample on ice in a cooler. Samples can be stored on ice (∼4 °C) or at room temperature, depending on the laboratory specifications for analysis. USGS Reston Groundwater Dating Laboratory recommends leaving samples at room temperature as this prevents gas in the water samples from exsolving [17]. The University of Utah recommends storing samples on ice or in a refrigerator at °4C [21].

## Method validation

The distribution of sulfur hexafluoride concentration is normal for both traditional and new methods. The variances are equal according to the folded F method (F-statistic=7.54, *p* < 0.0001). Therefore, comparison of means using a 2-sample *t*-test is an appropriate way to measure if the new and traditional methods give statistically significant differences in sulfur hexafluoride mean concentration. The results show there is a statistically significant difference between the new and traditional method means (µ= 0.16 fmol/L, t-statistic for new method=5.81, *p* < 0.0001; µ= 0.30 fmol/L, t-statistic for traditional method=4.00, *p* = 0.0008). Specifically, the new method mean (µ=0.16 fmol/L) is 0.14 fmol/L lower than the traditional method mean (0.30 fmol/L), indicating that new methods give an average lower-level detection than traditional methods. If this comparative methods analysis were duplicated, a larger dataset should be used to analyze the results.

**Fig. 6:**
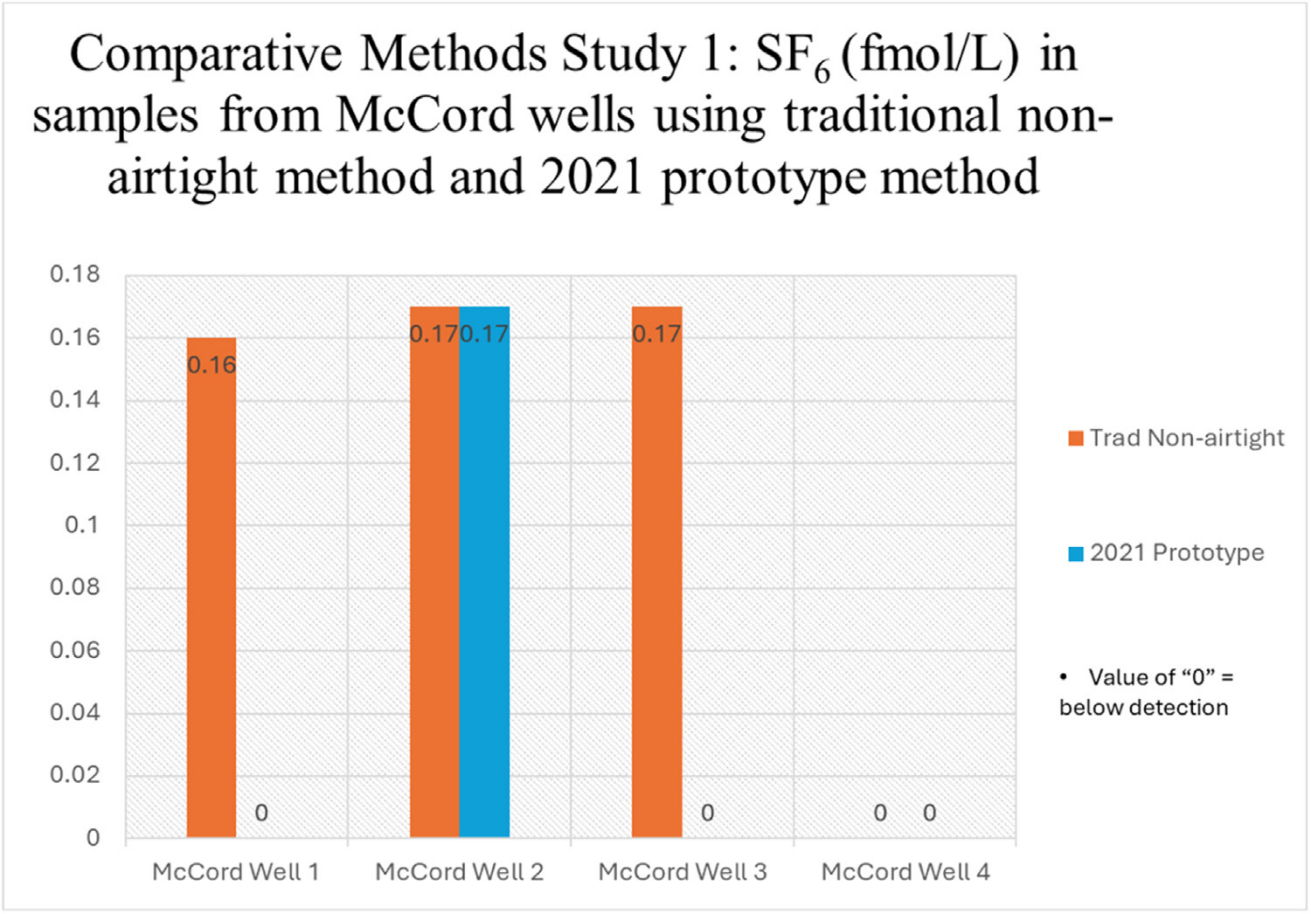
Comparative methods study using traditional USGS non-airtight method (Fig. 3A) and 2021 Prototype method (Fig. 3C, 4A).

**Fig. 7:**
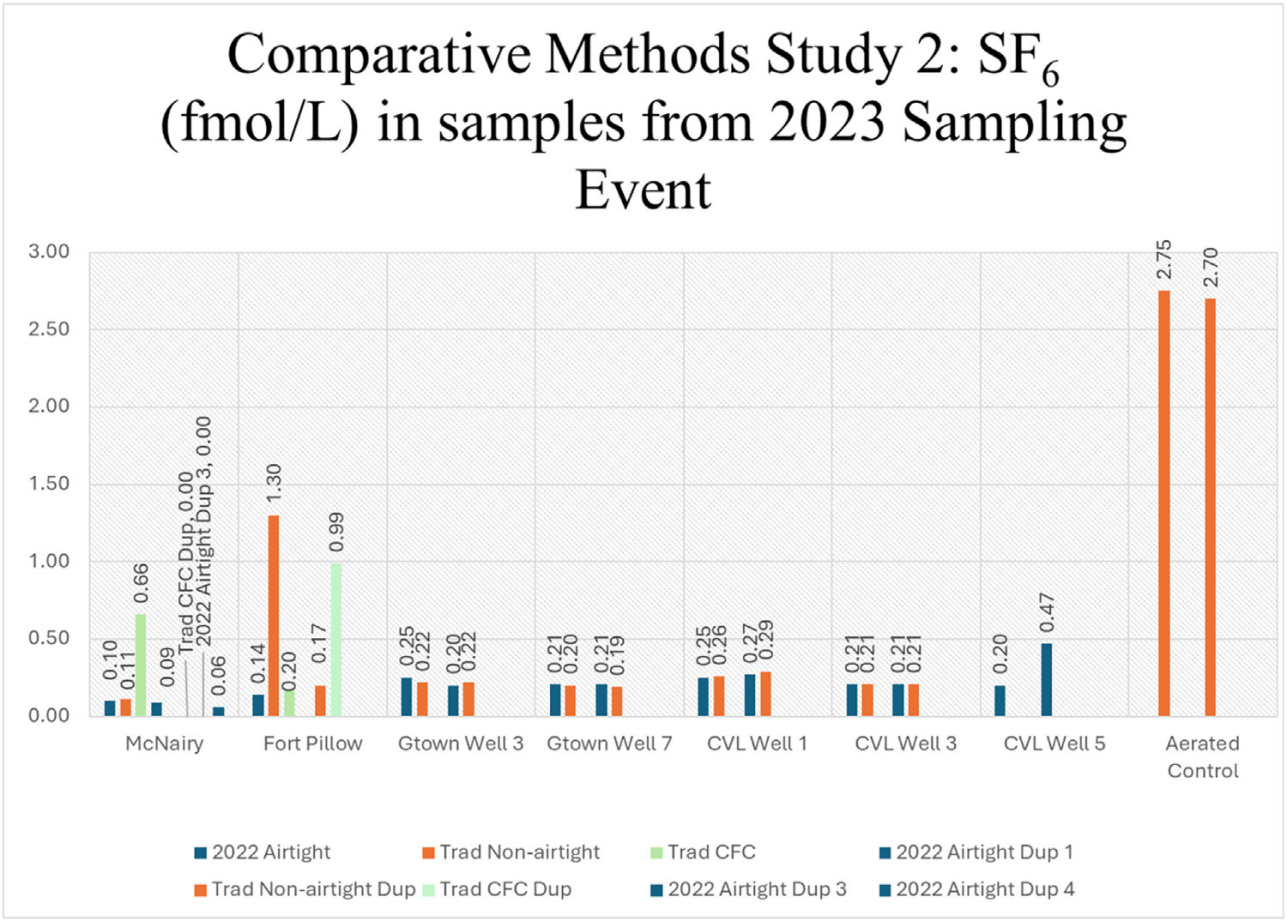
Comparative methods study using traditional USGS non-airtight method (Fig. 3A), traditional USGS CFC “air-sensitive” method (Fig. 3B), and 2022 airtight method (Fig. 3D, 4B).

Fig. 6, Fig. 7 summarize the results of the comparative methods studies performed between new and traditional methods. Additional data including well depth, depth to screen, distance to transferomers, and ^3^H concentrations for each sampled well are included in the supplemental materials section of this article. The 2021 airtight design returns results of 0.00 fmol/L for McCord Wells 1 and 3 while the traditional non-airtight design returns results of 0.16 fmol/L (McCord Well 1) and 0.17 fmol/L (McCord Well 3) (Fig. 6). This indicates that the 2021 airtight design plays a significant role in eliminating concentrations of SF_6_. McCord Well 4 SF_6_ concentrations were below detection for both methods. Tritium results for McCord Well 1,3, and 4 are between 0.03–0.18 TU, which is consistent with background levels [17]. The ^3^H results suggest that McCord wells 1,3, and 4 should also have background SF_6_ values. Any detection from the non-airtight sampling method for the McCord Wells 1 and 3 is attributed to modern-air contamination introduced during sampling, or sampling error.

McCord Well 2 returned 0.17 fmol/L with both 2021 and traditional non-airtight methods, respectively, which is inconsistent with 0.03 TU ^3^H result. This suggests that the 2021 method design was not completely airtight or that a small component of modern water is present in the sample. The 2021 method design contained permeable materials, such as silicone, which were removed in the 2022 airtight design. The 2021 design is inexpensive but is probably not suitable for precise sampling. Overall, the results from McCord Wells 1, 3, and 4 suggest that the 2021 design reduced sampling-related contamination from SF_6_ in modern air and warranted further improvement in the 2022 airtight design.

The 2022 airtight design shows very low SF_6_ values for the McNairy well and the Fort Pillow well, with all duplicates within ± 0.03 fmol/L of the background 0.02–0.09 fmol/L SF_6_ value if analyzed within a month of the sampling date and within ±0.05 fmol/L if analyzed within ten months of the sample date (Fig. 7). This was the expected result for these wells as they are not likely to have modern water leakage due to their screen depth of 400–790 m below land surface. The McNairy tritium value is 0.00 TU (previous tritium value for this well in 2021 was 0.05 TU [15]), which agrees with the low-level SF_6_ detection found using the 2022 airtight method. Although the traditional air-sensitive CFC method shows one sample with SF_6_ below detection, the duplicates do not reproduce this result, showing a value 0.90 fmol/L higher than background SF_6_ in the Fort Pillow well and 0.57 fmol/L higher in the McNairy well. The traditional non-airtight method is within ± 0.02 fmol/L of background SF_6_ in the McNairy well, but it does not show reproducibly low values in the Fort Pillow well, where concentrations are 0.08–1.21 fmol/L higher than background SF_6_ values. Current tritium values were sampled for the Fort Pillow in prior research [15] which agrees with background SF_6_ results. The results from these tests show the 2022 airtight design improved reproducibility and is better suited for eliminating false positives (detectable SF_6_ where one would not expect it to be present) than either traditional method. The use of multiple labs further confirmed that the 2022 airtight design provided superior results and that the concerns regarding non-reproducibility with traditional sampling methods in an urban environment are not related to a specific analytical lab.

The Memphis aquifer wells in Germantown and Collierville showed comparable above-background SF_6_ results with the traditional non-airtight method and the 2022 airtight method for all wells sampled, except in Collierville Well 5, where a traditional non-airtight sample was not taken (Fig. 7). Tritium values for Germantown and Collierville wells are consistent with slightly above background levels for all wells except Collierville Well 3. Given the more suburban location of Collierville wells, the absence of nearby sources of SF_6_ (1.5–4 km away), and the proximity to the unconfined region of the Memphis aquifer, the non-background SF_6_ (higher than 0.09 fmol/L) from these wells likely reflect modern water mixing with older Memphis aquifer water. No comparable traditional non-airtight data are available for Collierville Well 5, but disparity in results from other Collierville wells sampled with the 2022 airtight design suggests that great care must be taken in construction of the airtight sampling bottles and in minimizing analytical error. Germantown Wells 3 and 7, on the other hand, are 0.1–0.5 km from pole-mounted distribution transformers. Comparable results in Germantown Well 3 and Well 7 between the 2022 airtight and traditional non-airtight method indicate that pole-mounted distribution transformers do not present a problem with sampling in this locality.

The observation of non-background SF_6_ values in the non-airtight samples from McCord well field, McNairy, and Fort Pillow wells may be explained by modern water leakage and by well location within 0.5 km of high-capacity electrical transformers near pumping stations and/or distribution transformers if they contain SF_6_ rather than transformer oil (see supplemental materials for distances to electrical transfomers for each sampled well). Non-background SF_6_ values in the non-airtight and 2022 airtight design samples from the Memphis aquifer production wells in Germantown and Collierville wells are more likely explained by modern water recharge or leakage into the aquifer from local breaches in the confining unit. A detailed analysis of all possible sources of SF_6_ is provided in [8].

Sulfur hexafluoride is a dense gas that diffuses slowly in ambient air into the upper atmosphere via emission from electrical transformers [3]. Inconsistent SF_6_ concentrations in water samples have been found near urban wellfields [3]. It is possible that non-background SF_6_ concentrations could form by interaction with SF_6_-laden air during non-airtight sampling near electrical transformers since these are the major emitters of this gas. The results from sampling with the 2022 airtight method and supporting results from the 2021 prototype method suggest that the newly developed airtight method may reduce the effect of modern-air contamination during SF_6_ sampling of groundwater.

## Limitations

Limitations to the design of the 2022 Airtight method include the design and manufacture of an evacuation and nitrogen purge manifold setup that can be monitored with a manometer or pressure gauge. This setup requires additional cost and construction that may not be feasible for researchers. Construction of flasks requires use of a tightening zip tie tool. If the flask is not tightened adequately, it cannot be considered airtight, and must be tested to hold a pressure of 2–15 mmHg over 24 h before being used. Shipping to analytic laboratories is another additional cost and may cause breakage of flasks, rendering results useless. Great care must be taken during construction of flasks, storage, and shipment to avoid unecessary loss of samples.

## CRediT author statement

**Raka Sunderland:** Conceptualization, Data Curation, Methodology, Writing-Reviewing and Editing. **Dan Larsen:** Writing-Reviewing and Editing. **Scott Schoefernacker:** Writing-Reviewing and Editing. **Deborah Leslie:** Writing-Reviewing and Editing.

## Declaration of competing interest

The authors declare that they have no known competing financial interests or personal relationships that could have appeared to influence the work reported in this paper.

## Data Availability

Data will be made available on request.

## Appendix Supplementary materials

Image, application 1
