# Supplementary material for: A new airtight sampling method for sulfur hexafluoride (SF6) in groundwater
Source: MethodsX. 2024 Dec 18;14:103120. doi: 10.1016/j.mex.2024.103120 (PMC11732182; doi:10.1016/j.mex.2024.103120)
Supplement: Supplementary file 1 [file mmc1.docx]

**Supplemental Materials**

Table A: Comparison of SF_6_ values when using the traditional non-airtight method (Figure 3A) and 2021 prototype method (Figure 3C, 4A).

| Sampling  date | Sampling method | Sample well | Well Depth (m) | Depth to Screen (m) | Well Conditions | Distance to Nearest Local SF_6_  Source (km) | SF_6_ (fmol/L) | ^3^H (TU) |
| --- | --- | --- | --- | --- | --- | --- | --- | --- |
| 03/02/22 | Trad non-airtight | McCord Well 1 | 190 | 158 | Confined | 0.06 | 0.16 | 0.11 |
| 03/02/22 | 2021 Airtight | McCord Well 1 | 190 | 158 | Confined | 0.06 | 0.00 | 0.11 |
| 03/02/22 | Trad non-airtight | McCordWell 2 | 192 | 162 | Confined | 0.37 | 0.17 | 0.03 |
| 03/02/22 | 2021 Airtight | McCord Well 2 | 192 | 162 | Confined | 0.37 | 0.17 | 0.03 |
| 02/28/22 | Trad non-airtight | McCord Well 3 | 183 | 152 | Confined | 0.30 | 0.17 | 0.18 |
| 02/28/22 | 2021 Airtight | McCord Well 3 | 183 | 152 | Confined | 0.30 | 0.00 | 0.18 |
| 02/28/22 | Trad airtight | McCord Well 4 | 141 | 110 | Confined | 1.37 | 0.00 | 0.18 |
| 02/28/22 | 2021 Airtight | McCordWell 4 | 141 | 110 | Confined | 1.37 | 0.00 | 0.18 |

Table B: Comparison of SF_6_ and ^3^H values when using traditional air-sensitive CFC (Figure 3B), traditional non-airtight (Figure 3A), and 2022 airtight method (Figure 3D, Figure 4B) for the McNairy well and Fort Pillow production well.

| Sample date | Lab Analysis Location | Elapsed Time from Sampling to Analysis  (months) | Sampling  method | Sample well | Well Depth (m) | Depth to Screen  (m) | Distance to Local SF_6_ Source (km) | ^3^H  (TU) | SF_6_  (fmol/L) |
| --- | --- | --- | --- | --- | --- | --- | --- | --- | --- |
| 04/20/23 | USGS | 1 | Traditional CFC | McNairy | 792 | 199 | 0.30 | 0.00 | 0.66 |
| 04/20/23 | Utah | 10 | Traditional  CFC duplicate | McNairy | 792 | 199 | 0.30 | 0.00 | 0.00 |
| 04/20/23 | Utah | 10 | Traditional, non-airtight | McNairy | 792 | 199 | 0.30 | 0.00 | 0.11 |
| 04/20/23 | Utah | 10 | 2022 Airtight | McNairy | 792 | 199 | 0.30 | 0.00 | 0.10 |

*(Table B cont’d)*

| Sample date | Lab Analysis Location | Elapsed Time from Sampling to Analysis  (months) | Sampling  method | Sample well | Well Depth (m) | Depth to Screen  (m) | Distance to Local SF_6_ Source (km) | ^3^H  (TU) | SF_6_  (fmol/L) |
| --- | --- | --- | --- | --- | --- | --- | --- | --- | --- |
| 04/20/23 | Utah | 10 | 2022  Airtight  duplicate | McNairy | 792 | 199 | 0.30 | 0.00 | 0.09 |
| 04/20/23 | Utah | 10 | 2022  Airtight duplicate | McNairy | 792 | 199 | 0.30 | 0.00 | 0.00 |
| 04/20/23 | USGS | 1 | 2022 Airtight duplicate | McNairy | 792 | 199 | 0.30 | 0.00 | 0.06 |
| 04/25/23 | USGS | 1 | Traditional  non-airtight | Fort Pillow | 354 | 321 | 0.30 | N/A | 1.30 |
| 04/25/23 | Utah | 10 | Traditional, non-airtight duplicate | Fort Pillow | 354 | 321 | 0.30 | N/A | 0.20 |
| 04/25/23 | Utah | 10 | Traditional, CFC | Fort Pillow | 354 | 321 | 0.30 | N/A | 0.17 |
| 04/25/23 | Utah | 10 | Traditional, CFC duplicate | Fort Pillow | 354 | 321 | 0.30 | N/A | 0.99 |
| 04/25/23 | Utah | 10 | 2022  Airtight | Fort Pillow | 354 | 321 | 0.30 | N/A | 0.14 |

Table C: Comparison of SF_6_ and ^3^H values when using traditional non-airtight (Figure 3A), and 2022 airtight method (Figure 3D, Figure 4B) for Germantown (Gtown) and Collierville wells.

| Sample date | Lab Analysis Location | Elapsed Time from Sampling to Analysis  (months) | Sampling  Method | Sample well | Well Depth (m) | Depth to Screen (m) | Distance to Local SF_6_ Source (km) | Well Conditions | ^3^H  (TU) | SF_6_  (fmol/L) |
| --- | --- | --- | --- | --- | --- | --- | --- | --- | --- | --- |
| 11/09/23 | USGS | 1 | Traditional non-airtight | Gtown  Well 3 | 78 | 63 | 0.47 | Confined | 0.18 | 0.22 |
| 11/09/23 | USGS | 1 | Traditional non-airtight duplicate | Gtown  Well 3 | 78 | 63 | 0.47 | Confined | 0.18 | 0.22 |
| 11/09/23 | USGS | 1 | 2022 Airtight | Gtown  Well 3 | 78 | 63 | 0.47 | Confined | 0.18 | 0.25 |
| 11/09/23 | USGS | 1 | 2022 Airtight duplicate | Gtown  Well 3 | 78 | 63 | 0.47 | Confined | 0.18 | 0.20 |
| 11/09/23 | USGS | 1 | Traditional non-airtight | Collierville  Well 1 | 83 | 59 | 2.70 | Partially confined | 0.19 | 0.26 |
| 11/09/23 | USGS | 1 | Traditional non-airtight duplicate | Collierville  Well 1 | 83 | 59 | 2.70 | Partially confined | 0.19 | 0.29 |
| 11/09/23 | USGS | 1 | 2022 Airtight | Collierville  Well 1 | 83 | 59 | 2.70 | Partially confined | 0.16 | 0.25 |
| 11/09/23 | USGS | 1 | 2022 Airtight duplicate | Collierville  Well 1 | 83 | 59 | 2.70 | Partially confined | 0.16 | 0.27 |

*(Table C cont’d)*

| Sample date | | Lab Analysis Location | Elapsed Time Sampling to Analysis  (months) | Sampling  method | Sample well | | | Well Depth (m) | Depth to Screen (m) | | | Distance to Local SF_6_ Source (km) | | Well Conditions | | ^3^H  (TU) | SF_6_  (fmol/L) |
| --- | --- | --- | --- | --- | --- | --- | --- | --- | --- | --- | --- | --- | --- | --- | --- | --- | --- |
| 11/14/23 | USGS | 1 | Traditional non-airtight | | Collierville  Well 3 | 85 | | | 70 | 1.83 | | Confined | | 0.06 0.21 | | |  |
| 11/14/23 | USGS | 1 | Traditional non-airtight duplicate | | Collierville  Well 3 | 85 | | | 70 | 1.83 | | Confined | | 0.06 0.21 | | |  |
| 11/14/23 | USGS | 1 | 2022 Airtight | | Collierville  Well 3 | 85 | | | 70 | 1.83 | | Confined | | 0.06 0.21 | | |  |
| 11/14/23 | USGS | 1 | 2022 Airtight duplicate | Collierville  Well 3 | | | 85 | 70 | | | 1.83 | | Confined | | 0.06 | 0.21 |  |
| 11/14/23 | USGS | 1 | Traditional non-airtight | Gtown  Well 7 | | | 78 | 63 | | | 0.13 | | Confined | | 0.13 | 0.20 |  |
| 11/14/23 | USGS | 1 | Traditional non-airtight duplicate | Gtown  Well 7 | | | 78 | 63 | | | 0.13 | | Confined | | 0.13 | 0.19 |  |
| 11/14/23 | USGS | 1 | 2022 Airtight | Gtown  Well 7 | | | 78 | 63 | | | 0.13 | | Confined | | 0.13 | 0.21 |  |
| 11/14/23 | USGS | 1 | 2022 Airtight duplicate | Gtown  Well 7 | | | 78 | 63 | | | 0.13 | | Confined | | 0.13 | 0.21 |  |
